# Field performance of ultrasensitive and conventional malaria rapid diagnostic tests in southern Mozambique

**DOI:** 10.1186/s12936-020-03526-9

**Published:** 2020-12-07

**Authors:** Beatriz Galatas, Alfredo Mayor, Himanshu Gupta, Núria Balanza, Ihn Kyung Jang, Lidia Nhamussua, Wilson Simone, Pau Cisteró, Arlindo Chidimatembue, Humberto Munguambe, Francisco Saúte, Pedro Aide, Quique Bassat

**Affiliations:** 1 ISGlobal, Hospital Clínic - Universitat de Barcelona, Barcelona, Spain; 2 Centro de Investigação em Saúde de Manhiça, Maputo, Mozambique; 3 CIBER Epidemiología y Salud Pública (CIBERESP), Madrid, Spain; 4 PATH, Seattle, USA; 5 National Institute of Health, Ministry of Health, Maputo, Mozambique; 6 ICREA, Pg. Lluís Companys 23, 08010 Barcelona, Spain; 7 Pediatric Infectious Diseases Unit, Pediatrics Department, Hospital Sant Joan de Déu (University of Barcelona), Barcelona, Spain; 8 Present Address: Department of Infection Biology, Faculty of Infectious and Tropical Diseases, London School of Hygiene and Tropical Medicine, London, UK

**Keywords:** Malaria, Diagnostics, Rapid diagnostic test (RDT), Ultrasensitive, Low density parasitaemia, HRP2, *Plasmodium falciparum*, Mozambique

## Abstract

**Background:**

An ultrasensitive malaria rapid diagnostic test (RDT) was recently developed for the improved detection of low-density *Plasmodium falciparum* infections. This study aimed to compare the diagnostic performance of the *Pf*HRP2-based Abbott Malaria Ag *P. falciparum* ultrasensitive RDT (uRDT) to that of the conventional SD-Bioline Malaria Ag *P. falciparum* RDT (cRDT) when performed under field conditions.

**Methods:**

Finger-prick blood samples were collected from adults and children in two cross-sectional surveys in May of 2017 in southern Mozambique. Using real-time quantitative PCR (RT-qPCR) as the reference method, the age-specific diagnostic performance indicators of the cRDT and uRDT were compared. The presence of histidine-rich protein 2 (HRP2) and *Plasmodium* lactate dehydrogenase (pLDH) antigens was evaluated in a subset from dried blood spots by a quantitative antigen assay. *pfhrp2* and *pfhrp3* gene deletions were assessed in samples positive by RT-qPCR and negative by both RDTs.

**Results:**

Among the 4,396 participants with complete test results, the sensitivity of uRDTs (68.2; 95% CI 60.8 to 74.9) was marginally better than that of cRDTs (61.5; 95% CI 53.9 to 68.6) (p-value = 0.004), while the specificities were similar (uRDT: 99.0 [95% CI 98.6 to 99.2], cRDT: 99.2 [95% CI 98.9 to 99.4], p-value = 0.02). While the performance of both RDTs was lowest in ≥ 15-year-olds, driven by the higher prevalence of low parasite density infections in this group, the sensitivity of uRDTs was significantly higher in this age group (54.9, 95% CI 40.3 to 68.9) compared to the sensitivity of cRDTs (39.2, 95% CI 25.8 to 53.9) (p-value = 0.008). Both RDTs detected *P. falciparum* infections at similar geometric mean parasite densities (112.9  parasites/μL for uRDTs and 145.5 parasites/μL for cRDTs). The presence of HRP2 antigen was similar among false positive (FP) samples of both tests (80.5% among uRDT-FPs and 84.4% among cRDT-FPs). Only one false negative sample was detected with a partial *pfhrp2* deletion.

**Conclusion:**

This study showed that the uRDTs developed by Abbott do not substantially outperform SD-Bioline *Pf* malaria RDTs in the community and are still not comparable to molecular methods to detect *P. falciparum* infections in this study setting.

## Background

Despite control and elimination efforts worldwide, malaria remains a major global health problem. This mosquito-borne disease caused by *Plasmodium* parasites accounted for an estimated 228 million cases and 405,000 deaths in 2018, most of them circumscribed to sub-Saharan Africa and concentrated in the under-five years of age and pregnant populations [1]. The World Health Organization (WHO) Global Technical Strategy for malaria (2016–2030) promotes that all malaria-endemic countries should ensure universal access to malaria prevention, diagnosis and treatment while transforming their malaria surveillance systems into core interventions [2]. Therefore, the accurate detection of malaria infections is essential to provide high-quality case management to all malaria infected patients, as well as to inform surveillance systems to allow monitoring progress and guiding where to target interventions.

Microscopic visualization of peripheral blood smears has traditionally been the most commonly used method to confirm the presence of *Plasmodium* parasites, but this changed with the appearance of malaria rapid diagnostic tests (RDTs) in the early 2000s [1]. Malaria RDTs are a simple, rapid, cheap and field-deployable way to accurately identify a malaria infection at the point of care, being especially useful in low-resource and rural settings without access to laboratory facilities. Their use has sharply increased in the last years and an estimated 412 million tests were sold globally in 2018 [1]. These RDTs are immunochromatographic lateral flow devices capable of detecting one or more specific *Plasmodium* proteins in a small volume of blood (typically a single drop, which can be obtained by finger-prick). There are various commercially available tests that detect different parasite molecules produced during the erythrocytic cycle, namely the *Plasmodium falciparum* (*Pf*) histidine-rich protein 2 (HRP2), the *Plasmodium* genus or species-specific lactate dehydrogenase (pLDH) and the *Plasmodium* genus or species-specific aldolase [3].

HRP2-based RDTs are the most widely used across malaria-endemic Africa [1]. However, their performance is affected by a series of factors [4, 5]. False positive results can be due to: (i) HRP2 persistence in the bloodstream after infection clearance (and thus, are not technically false positives), which has been reported to last for several weeks (up to 4–6 weeks) after treatment [6, 7]; and to a lesser extent (ii) presence of autoantibodies and other infections [8]. False negative results can be caused by: (i) parasite *pfhrp2* and *pfhrp3* gene deletions, which cause absence of *Pf*HRP2 expression and have been increasingly observed throughout Latin America and Africa [9, 10]; (ii) very high parasite densities (i.e. prozone effect) [11]; as well as (iii) low-density parasite infections under the RDT detection threshold capacity. Given that regular RDTs have a detection threshold of ~ 100 parasites/μL (0.002% parasitaemia) [12], this is of particular relevance in malaria endemic areas where a large proportion of malaria infected individuals, usually afebrile, have parasite densities below the RDTs threshold of detection [13–15]. Thus, their infections remain undetected and hence uncleared for longer periods of time, representing a potential—yet poorly understood—reservoir of infection in endemic areas [16]. Missing them may hinder efforts to reduce transmission and leads to inaccurate burden estimations.

Polymerase chain reaction (PCR) assays have great analytical performance and can detect very low parasitaemias (as low as < 1 parasites/μL) [17, 18], but they are impractical for field use and their high cost keep them inaccessible in malaria endemic settings. The renewed interest in malaria elimination in the past decade has instigated discussions around the need for more sensitive malaria RDTs that can detect lower-level parasite densities than conventional RDTs (cRDTs) in the community, to be used mainly in efforts aiming to interrupt transmission. In this context, the Abbott (previously Alere™) Malaria Ag *P. falciparum* ultrasensitive RDT (uRDT) was developed. This is an HRP2-based test that has a tenfold lower limit of detection in vitro (40–125 pg/ml of HRP2) when compared to the commonly used SD-Bioline Malaria Ag *P. falciparum* RDT (800–1000 pg/ml) [19] and was prequalified by the WHO in 2019. It has the same immunochromatographic cassette platform, requires the same blood volume (5μL) and takes only 5 min longer (20 instead of 15) to obtain a result compared to the SD-Bioline RDT (cRDT), but has worse storage stability (30 °C versus 40 °C) [19].

Previous studies have evaluated the diagnostic performance of uRTDs in different transmission settings, populations and use-case scenarios, with the aim of informing malaria elimination strategies, or improving case management in the general population and among pregnant women. Several studies evaluated uRDT performance in cross-sectional surveys using stored frozen-thawed blood samples or running tests under laboratory conditions. These studies have shown a great variation in uRDT sensitivities when compared to PCR (from 27 to 84) [20–23], but results are not comparable to those obtained under field conditions. Assessment of the performance of the uRDTs in cross-sectional surveys conducted in The Gambia, Myanmar and Cambodia indicate that uRDTs outperforms cRDTs in different magnitudes when used to test individuals in the community. uRDT sensitivity estimates also varied across studies (from 35 to 60, when a PCR assay was considered the gold-standard), but all studies consistently reported that uRDTs miss a high percentage of infections detected by molecular methods [23–26]. Studies evaluating the potential role of uRDTs in case management found no differences in the performance of uRDTs and cRDTs for clinical malaria diagnosis among non-pregnant patients [27]. Finally, a similar or higher performance of uRDTs compared to cRDTs has been reported in pregnant women when tested using retrospectively collected peripheral or placental blood samples [28–30].

Mozambique is one of the countries with the highest malaria burden worldwide [1]. cRDTs were introduced in 2007 and they were rolled out at the national level in 2010. *Pf*HRP2-based RDTs are used for malaria diagnosis in the country, as *P. falciparum* is the major cause of malaria and there is a low prevalence of parasites with *pfhrp2* and *pfhrp3* gene deletions [31, 32]. In this study, the diagnostic performance of the *Pf*HRP2-based Abbott Malaria Ag *P. falciparum* uRDT and the SD-Bioline Malaria Ag *P. falciparum* cRDT were evaluated against RT-qPCR in children and adults who participated in two cross-sectional studies in the districts of Manhiça and Magude in Maputo province, southern Mozambique. The parasite density distributions of the detected infections were explored, and their performance compared, based on the presence of HRP2 antigens and *pfhrp2* and *pfhrp3* gene deletions.

## Methods

### Study site

**Fig. 1:**
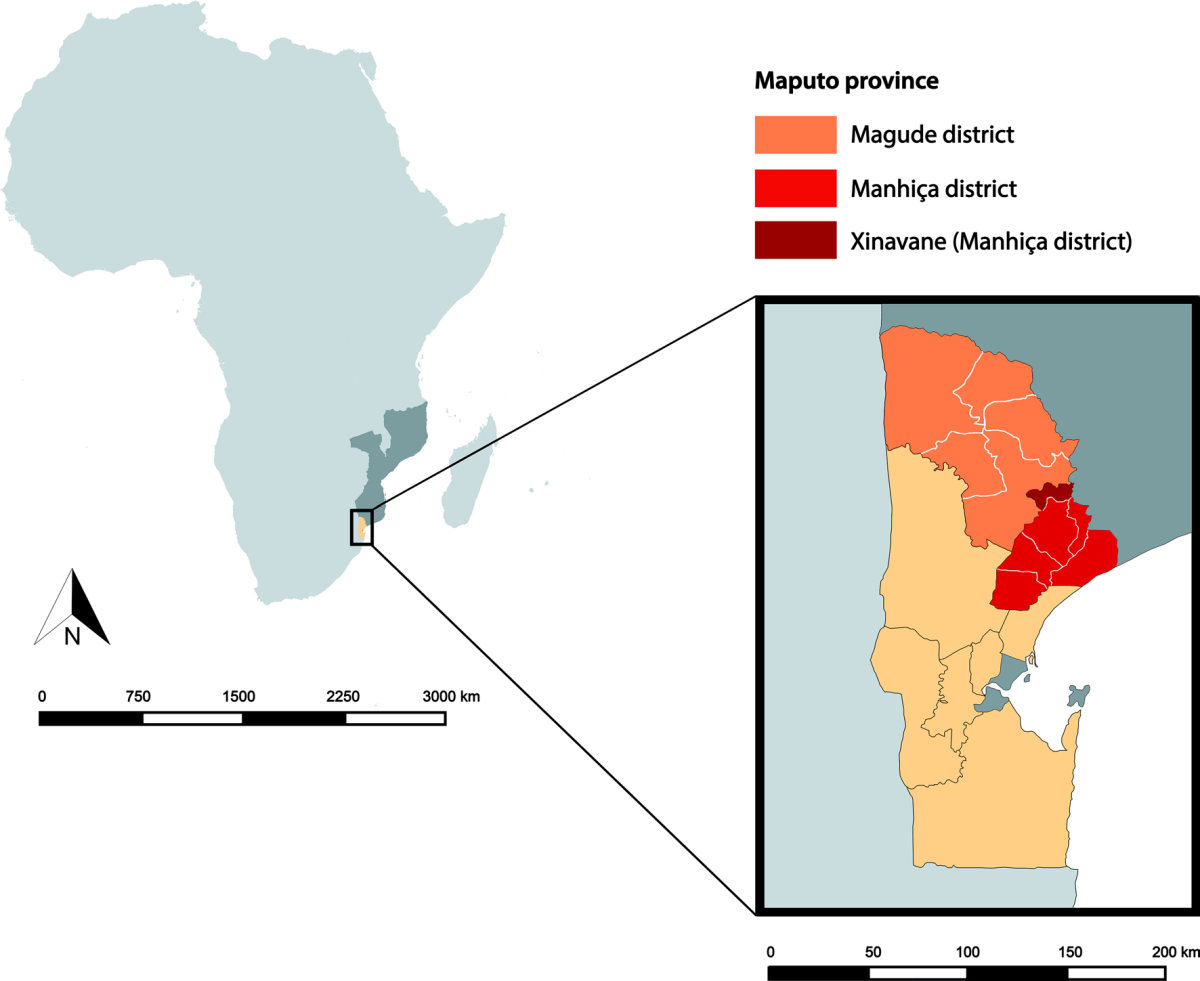
Map of Magude district, Manhiça district and Xinavane administrative post (Manhiça district); all located in Maputo Province, the southernmost province of Mozambique

The districts of Magude and Manhiça are located in Maputo province, southern Mozambique (Fig. 1). The district of Manhiça is a rural district of ~ 2300 km^2^ located 80 km north of Maputo City, the capital of Mozambique. Manhiça district is divided in six administrative posts: Maluana, Manhiça, Calanga, 3 de Fevereiro, Ilha Josina Machel and Xinavane. According to the national 2017 census, it has a population of ~ 208,000 inhabitants and ~ 50,000 households [33]. The district of Magude borders with Manhiça on the north-west and is the northernmost district of Maputo province. It is divided in five administrative posts: Magude Sede, Motaze, Panjane, Mahele and Mapulanguene. According to the national 2017 census, it has a population of ~ 64,000 inhabitants and ~ 15,000 households and it has ~ 7000 km^2^ [33]. The Manhiça Health Research Center (CISM) expanded its presence to Magude in 2014, in the context of a malaria elimination feasibility project planned for the district for the period of 2015–2019 [34].

*Plasmodium falciparum* malaria transmission is perennial in the area but with a peak between November and April, coinciding with the rainy season. The incidence of passively reported malaria cases during the transmission season of the study (July 2016 to June 2017) was 186 cases per 1000 in Manhiça and 75 cases per 1000 in Magude [35] *Anopheles funestus* is the most abundant vector responsible for the majority of transmission, followed by *Anopheles arabiensis* [36, 37]. According to national case management guidance, all febrile patients are tested for malaria through *Pf*HRP2-based cRDTs and uncomplicated malaria cases are offered treatment with artemether–lumefantrine (AL), the first-line treatment in Mozambique. Prior to this study, vector control measures in both districts consisted of routine long-lasting insecticidal net (LLIN) distributions to pregnant women attending the ANCs and children attending routine immunization services, as well as mass LLIN campaigns. Indoor residual spraying (IRS) has also been conducted in both districts through district-wide campaigns or focalized to the highest burden areas.

### Study design

Two age stratified cross-sectional surveys with an over-representation of children were conducted in the districts of Magude and Manhiça at the end of the malaria transmission season, in May 2017. The cross-sectional survey conducted in Manhiça covered the original study area of the demographic surveillance system (DSS) established by CISM in Manhiça, which includes the entire district except for the administrative posts of Xinavane and Calanga [38]. This survey was planned as part of the annual malaria surveys that started in 2010 to monitor malaria prevalence and vector control use in the district. The second survey covered the entire district of Magude and the administrative post of Xinavane (Manhiça), and it was aimed to measure infection prevalence after the first phase of a malaria elimination project being implemented in the area since 2015 [34].

In both surveys, a simple random sample of participants was selected per age group from the census databases available from Manhiça and Magude, collected and updated through the demographic surveillance platforms established in both districts [38, 39]. The age groups used to stratify the sample differed according to the district. In Manhiça, the age groups were: < 1, 1–< 2, 2–< 3, 3–< 4, 4–< 5, 5–< 10, 10–< 20, 20–< 40, 40–< 60, > 60. In Magude district and Xinavane, the age groups were: < 6 months, 6 months–< 2, 2–< 5, 5–< 15, > 15. The sample size for Manhiça and Xinavane was calculated to detect a prevalence of parasitaemia of 50% (conservative estimate with maximum imprecision) with a precision of ± 10% and a confidence coefficient of 95%. On the other hand, the sample size of the survey in Magude was calculated for each specific age group to detect a reduction in malaria prevalence of 90% with regards to that detected on the preceding year, with a power of 90% and a significance level of 5% [35].

### Field and laboratory procedures

RDTs were performed under field-conditions. Participants’ fingers were disinfected and pricked by trained workers using the alcohol swabs and lancets provided by the manufacturer in the RDT kits. Approximately 5  μL of blood were collected using the manufacturer’s specimen collection inverted cups for the assessment of malaria infection with SD-Bioline RDT and Abbott RDT. Results were recorded for both tests after approximately 20 min. Individuals with a confirmed infection by either test were treated with AL (Coartem®) according to national guidelines.

A volume of approximately 30 μL of finger-pricked blood was collected using the specimen collection inverted cups provided in the RDT kits for the determination of parasitaemia on two thick (~ 10 μL each) and thin (~ 5 μL each) blood slides. Smears were air-dried, stained with Giemsa and examined using a light microscope fitted with a 100 × oil immersion lens and a 10 × eyepiece to quantify parasitaemia in CISM’s laboratory. Slides were read twice by two different qualified microscopists using the Lambaréné method [40] A third reading was performed by an additional microscopist in the case of discordant results. If the third reading was positive, the sample was considered positive. Samples with a negative third reading were considered negative.

Two Whatman 903™ filter papers with two 50 μL blood spots were also collected. During the field activities, filter papers and blood smears were placed in clean containers where the blood did not come into contact with other materials. Afterwards, they were wrapped in aluminum paper with silica gel and placed in − 20 °C. All filter papers and smears were labelled with a unique identification number for each study participant. If blood collection was challenging and only a small volume was obtained, RDTs and RT-PCR were prioritized over blood slides.

Real time quantitative PCR (RT-qPCR) analyses were conducted in the laboratory at CISM (Manhiça, Mozambique), with an estimated limit of detection of about 2–5 parasites/μL [41]. DNA was extracted from half of a 50 µL dried blood drop on Whatman 903™ filter paper by using a QIAamp DNA Mini kit (250) from QIAGEN, according to the manufacturer’s instructions and eluted in 100 μL of elution buffer. The ABI PRISM 7500 HT Real-Time System (Applied Biosystems) was used to amplify purified parasite DNA templates, using a previously described method that targets the *P. falciparum* 18S rRNA [41, 42] Parasitaemia in the clinical samples was quantified by extrapolation against the standard curve prepared from an in vitro culture of the 3D7 strain. Samples without amplification (Ct > 40) were considered negative. A negative control with no template DNA was run in all reactions.

The presence of HRP2 and two different pLDH (Pan specific pLDH [pLDH-Pan] and *P. falciparum* specific pLDH [pLDH–Pf]) antigens were estimated at PATH’s laboratories (Seattle, USA) through a Q-plex enzyme-linked immunosorbent assay (ELISA) from a subset of 223 samples comprised of samples positive by RT-qPCR and either cRDT or uRDT (N = 82), negative by all diagnostic forms (N = 45), as well as all samples positive by cRDT or uRDT but negative by RT-qPCR (N = 44) and samples positive by RT-qPCR but negative by both RDTs (N = 52). A small disc (6-mm) was punched out of each dried blood spot (DBS) and transferred to a well in a low-binding V-bottom 96-well plate (Thermo Scientific) with calibrator diluent (Quansys Bioscience, Logan, UT). The plate was incubated overnight in 4 °C and shaken for 1 h at room temperature. The eluates were tested in two replicates on Q-Plex Human Malaria Array (5-Plex, Quansys Biosciences) according to the manufacturer’s instructions [43], with one minor modification on the use of 4 × concentrated competitor. The positive and negative results were determined by the cutoff values which correspond to a lower 95% confidence bound of > 92% specificity from the receiver-operating characteristic (ROC) analysis performed using PCR-confirmed clinical samples (Jang et al. 2020; pers. commun.). Given that the HRP2-concentration levels in most samples were near the upper limit of quantification, parasite antigen data were used qualitatively to determine the presence or absence of antigens in relation to cRDT, uRDT and RT-qRTPCR results.

Finally, all samples that were positive by RT-qPCR but negative by both uRDT and cRDT (N = 55) were explored for parasite *pfhrp2* and *pfhrp3* gene deletions as described elsewhere [31]. First, to verify the presence of parasite DNA in the sample, a nested PCR targeting a single copy of the *k13* gene was performed [44]. Secondly, samples found positive for *k13* gene amplification were subjected to *pfhrp2* and *pfhrp3* PCRs, amplifying regions across exon 1 and 2 as well as only exon 2 [31]. Positive controls (laboratory-adapted culture lines 3D7 for both *pfhrp2* and *pfhrp3*) and negative controls (HB3 for *pfhrp3* and DD2 for *pfhrp2*) were simultaneously amplified. PCR products were visualized using 2% agarose (Invitrogen) and a UV trans-illuminator. Lastly, *pfhrp2* and *pfhrp3* deletions were considered if *k13* gene PCR was positive but PCRs for *pfhrp2* and *pfhrp3* failed to amplify the respective gene fragments [31].

### Data collection and management

Participants’ socio-demographic information, history of fever and malaria control and treatment measures used, as well as cRDT and uRDT results were collected during the field visits through an electronic questionnaire using the open access software REDCap [45, 46]. Results from all laboratory techniques (microscopy, RT-qPCR, ELISA, and *pfhrp2* and *pfhrp3* gene deletion assessments) were matched through the patients’ unique identifier to the field database.

Samples that did not have results for at least one of the main diagnostic tools (cRDT, uRDT and RT-qPCR) were excluded from this analysis. The distribution of characteristics was compared between individuals with (n = 3946) and without (n = 450) a microscopy diagnosis in order to evaluate the representativeness of the microscopy results. The characteristics of participants with a microscopy results were not significantly different from those without a microscopy result (all p > 0.05), and their information was, therefore, considered to be representative of the entire study sample.

### Data analysis

Data were analysed using Stata/IC 16 (Stata Corp, College Station, Texas, USA). Basic characteristics of participants were summarized using descriptive analyses. The pooled all-age prevalence of malaria measured by different diagnostic methods was calculated separately for each study area (Manhiça, Xinavane and Magude), taking into account the different age weights as per the study design and for the complete study sample (prior to the exclusion of participants for the diagnostic performance analysis, as described above). The percentage of positive samples per diagnostic type (microscopy, cRDT, uRDT, and RT-qPCR) was reported for the entire population and stratified by age groups (< 5, 5–< 15 and ≥ 15 years old).

The diagnostic performance of cRDT and uRDT were assessed using RT-qPCR as the gold standard. An infection was considered true positive (TP) if it was both RT-qPCR-positive and RDT-positive, true negative (TN) if it was both RT-qPCR-negative and RDT-negative, and false negative (FN) if RDT-negative and RT-qPCR-positive. RDT-positive and RT-qPCR-negative results were considered (and will be referred to as such hereafter) as false positives (FP), understood as samples without an active infection, despite acknowledging that these results may be detecting HRP2 antigenaemia from recently resolved infections, and thus may not technically be real false positives [20]. The association of age group and an RDT FP or FN result was explored calculating odds ratios (ORs) with logistic regression and estimating the strength of evidence with likelihood-ratio tests. The following performance indicators were calculated for cRDT and uRDT: sensitivity, specificity, positive predictive value (PPV), negative predictive value (NPV), positive likelihood ratio (LR+), negative likelihood ratio (LR-), diagnostic odds ratio (DOR) and area under the receiver operating characteristic curve (AUC) (Additional file 1: Table S1 presents the formulae used). Performance indicators were also calculated separately for the age groups < 5, 5–< 15 and ≥ 15 years old. Exact McNemar’s tests were used to compare performance indicators between RDTs for the entire population and for same age groups. Chi-squared tests were used to compare performance indicators between age groups within an RDT type.

Geometric mean parasite densities (GMPDs) and minimal and maximal parasitaemias were calculated for different cRDT and uRDT outcomes, for the entire population and stratified by age group. Parasite density distributions of infections detected by RT-qPCR, uRDT, cRDT and microscopy were also described using smoothing Kernel density estimations. HRP2, pLDH-Pan, pLDH-Pf and pLDH-Pv antigenic positivity proportions were calculated for the aforementioned subset of 223 samples stratified by RT-qPCR result. Chi-squared tests were used to compare antigen positivity between cRDT and uRDT. The proportion of infections with *pfhrp2* and *pfhrp3* gene deletions were calculated in the subset of both uRDT and cRDT FN samples.

As a secondary analysis, the performance of RDTs between febrile and afebrile individuals was also explored. Individuals were defined as febrile if their axillary temperature was ≥ 37.5 °C when measured during the household visit or reported having had fever in the preceding 24 h, and afebrile if their axillary temperature was < 37.5 °C without reporting having had fever in the preceding 24 h. The presence of fever among individuals with RT-qPCR or RDT positive results was measured, and a comparison of the parasite density distributions between febrile and afebrile cases made. uRDT and cRDT sensitivity and specificity were calculated for both groups, and comparisons were made using Chi-squared tests and McNemar’s tests as previously explained. Finally, diagnostic results were explored among the pregnant women found during the surveys.

### Ethical considerations

Study protocols were approved by CISM’s institutional ethics committee, Hospital Clínic of Barcelona’s Ethics Committee, and the Mozambican Ministry of Health National Bioethics Committee (reference numbers 143/CNBS/17 and 90/CNBS/16). Prior to their initiation, the surveys and their respective designs were communicated to the community leaders of Manhiça and Magude through the community outreach platforms established by CISM in both districts. Study results were shared with the community through the same channels. Written informed consent and oral assent (for 12 to 17 year-olds) was sought from all individuals, or parents/guardians if participants were younger than 18 years old.

## Results

### Study population characteristics

A total of 5342 participants were recruited from Manhiça (18.4%), Xinavane (9.2%) and Magude (72.4%) in May 2017. Of these, 946 samples (17.7%) were excluded from the analysis as they lacked results for one or both RDTs and/or RT-qPCR. Among the 4396 participants with complete test results, the median age was 4 years old (Interquartile range [IQR] = 3–10). Women accounted for 55.2% (2402/4353) of the study sample, among whom 21 reported to be pregnant. Only 0.5% (19/4128) of participants presented a body temperature ≥ 37.5 °C during testing. However, 8.5% (371/4387) reported having a febrile episode in the preceding 24 h. Within the previous month, 14.6% (640/4394) reported having a febrile episode and 6.5% (284/4342) reported taking anti-malarials. Additional file 1: Table S2 presents the detailed study population characteristics for the entire study sample and stratified by region.

### Malaria prevalence

The all-age weighted malaria prevalence by cRDT was 6.1% (95% confidence interval (CI) 4.2% to 8.7%) in Manhiça, 1.9% (95% CI 0.9% to 3.9%) in Xinavane and 2.6% (95% CI 2.0% to 3.4%) in Magude, while the weighted prevalence by uRDT in these same settings was 6.5% (95% CI 4.6% to 9.1%), 2.8% (95% CI 1.4% to 5.8%) and 3.2% (95% CI 2.3% to 4.0%), respectively. The same indicator measured by RT-qPCR was 7.9% (95% CI 5.5% to 11.3%) in Manhiça, 6.1% (95% CI 3.4% to 10.8%) in Xinavane and 4.5% (95% CI 3.6% to 5.6%) in Magude.

**Fig. 2:**
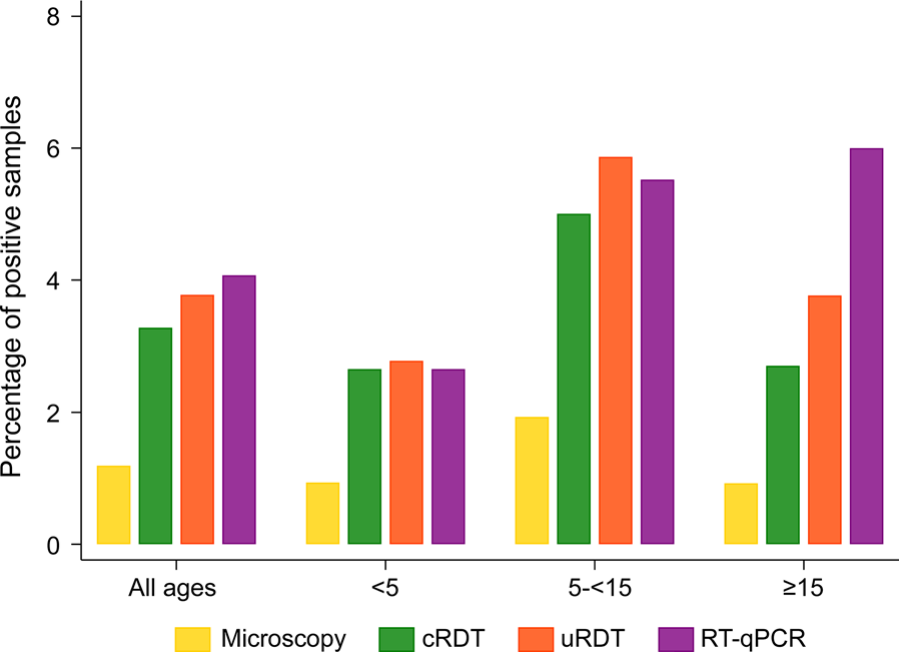
Percentage of infections detected per diagnostic tool, according to age group. All ages N = 4396 (microscopy N = 3946); < 5 years old N = 2373 (microscopy N = 2138); 5–< 15 years old N = 1159 (microscopy N = 1036); ≥ 15 years old N = 850 (microscopy N = 757)

Throughout all areas, there were 144 (3.3%) *P. falciparum* infections detected by cRDT, 166 (3.8%) by uRDT, and 179 (4.1%) by RT-qPCR (Table 1). Of the 3,946 individuals for whom blood slide results were available, there were 47 (1.2%) infections detected by light microscopy (Fig. 2). When stratifying by age, the differences in the percentage of samples positive by each diagnostic tool were intensified in the ≥ 15-year-old category (Fig. 2). In all age categories, microscopy detected a lower percentage of positive samples compared to the other diagnostic tools.

**Table 1:** cRDT and uRDT diagnostic classification using RT-qPCR as the gold-standard

cRDT	RT-qPCR			uRDT	RT-qPCR		
	+ve	−ve	Total		+ve	−ve	Total
+ ve	110	34	144	+ve	122	44	166
− ve	69	4183	4252	−ve	57	4173	4230
Total	179	4217	4396	Total	179	4217	4396

### Performance of cRDT and uRDT compared to RT-qPCR

**Fig. 3:**
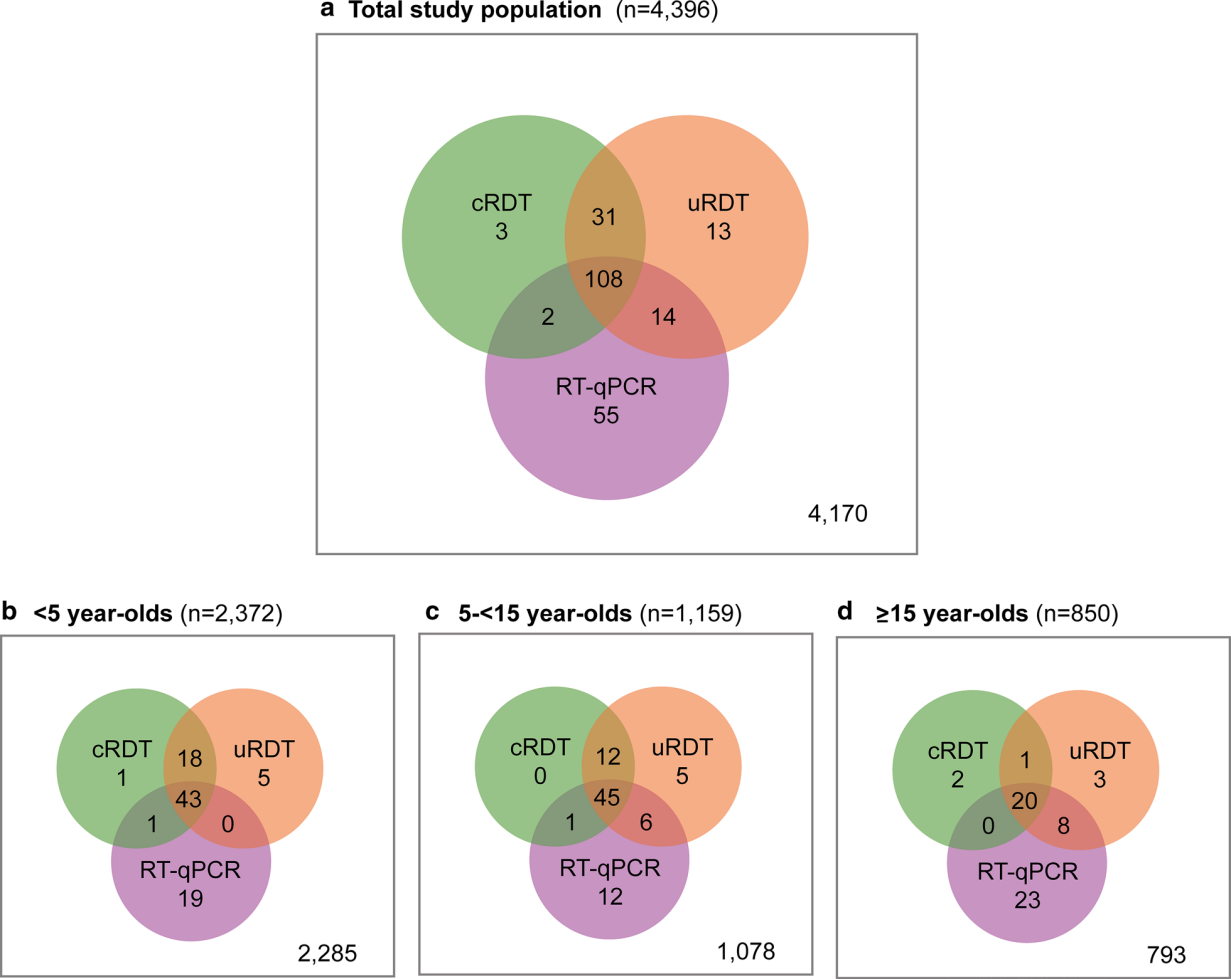
Venn diagrams of the distribution of positive results according to the diagnostic test used, in the entire population (**a**), in the < 5 year-olds (**b**), in the 5–15 year-olds (**c**) and in the ≥ 15 year-olds (**d**)

Considering RT-qPCR as the gold standard, cRDT misclassified 34 RT-qPCR-negative samples as positive (FP) and 69 RT-qPCR-positive samples as negative (FN), while uRDT misclassified 44 RT-qPCR-negative samples as positive and 57 RT-qPCR-positive samples as negative (Table 1). Figure 3 presents a detailed breakdown of samples per combination of diagnostic results. Most FP were concentrated among the < 15 year-olds (31/34 in cRDTs and 40/44 in uRDTS). The odds of having a FN result by either RDT was significantly higher among the eldest and, contrarily, the odds of being FP was higher among the youngest (Table 2).

**Table 2:** Logistic regression odd ratios of having a uRDT or cRDT false positive or negative result by age group

	n/N*	Odds Ratio	Likelihood-ratio test
uRDT false positives			
< 5	23/66	1	0.05
5–< 15	17/68	0.62 (0.30–1.32)	
≥ 15	4/32	0.27 (0.08–0.86)	
cRDT false positives			
< 5	19/63	1	0.19
5–< 15	12/58	0.60 (0.26–1.39)	
≥ 15	3/23	0.35 (0.09–1.31)	
uRDT false negatives			
< 5	20/2306	1	< 0.001
5–< 15	13/1091	1.38 (0.68–2.78)	
≥ 15	23/818	3.31 (1.81–6.05)	
cRDT false negatives			
< 5	19/2309	1	< 0.001
5–< 15	18/1101	2.00 (1.05–3.83)	
≥ 15	31/827	4.69 (2.64–8.36)	

^*^ The N includes all positive samples (for the false-positive analysis) and all negative samples (for the false-negative analysis)

The sensitivity of uRDTs was slightly higher (68.2; 95% CI 60.8 to 74.9) than that of cRDTs (61.5; 95% CI 53.9 to 68.6) (p-value = 0.004), while the specificity of both RDTs was very similar: 99.0 (95% CI 98.6 to 99.2) for uRDT and 99.2 (95% CI 98.9 to 99.4) for cRDT (p-value = 0.02). Table 3 presents the RDT-specific estimates of all performance indicators calculated for the study sample and stratified by age. The age-specific sensitivities decreased significantly with increasing age within each RDT (uRDT p-value = 0.02, cRDT p-value < 0.001), but were similar when compared between RDTs for the < 5 and 5 to 15-year-olds group. However, among > 15-year-olds, the sensitivity of uRDTs was substantially higher (54.9, 95% CI 40.3 to 68.9) than that of cRDTs (39.2, 95% CI 25.8 to 53.9) (p-value = 0.008). Specificity was very high (≥ 98.5) across all age groups for both RDTs (Table 3).

**Table 3:** cRDT and uRDT performance indicators using RT-qPCR as the gold-standard, overall and by age group

	RDT type	All ages (N = 4396)		< 5 years old (N = 2373)		5–15 years old (N = 1159)		≥ 15 years old (N = 850)	
		Estimate	(95% CI)	Estimate	(95% CI)	Estimate	(95% CI)	Estimate	(95% CI)
Sensitivity	cRDT	61.5	(53.9–68.6)	69.8	(57.0–80.8)	71.9	(59.2–82.4)	39.2	(25.8–53.9)
	uRDT	68.2	(60.8–74.9)	68.3	(55.3–79.4)	79.7	(67.8–88.7)	54.9	(40.3–68.9)
Specificity	cRDT	99.2	(98.9–99.4)	99.2	(98.7–99.5)	98.9	(98.1–99.4)	99.6	(98.9–99.9)
	uRDT	99.0	(98.6–99.2)	99.0	(98.5–99.4)	98.5	(97.5–99.1)	99.5	(98.7–99.9)
PPV	cRDT	76.4	(68.6–83.1)	69.8	(57.0–80.8)	79.3	(66.7–88.8)	87.0	(66.4–97.2)
	uRDT	73.5	(66.1–80.0)	65.2	(52.4–76.5)	75.0	(63.0–84.7)	87.5	(71.0–96.5)
NPV	cRDT	98.4	(98.0–98.7)	99.2	(98.7–99.5)	98.4	(97.4–99.0)	96.3	(94.7–97.4)
	uRDT	98.7	(98.3–99.0)	99.1	(98.7–99.5)	98.8	(98.0–99.4)	97.2	(95.8–98.2)
LR +	cRDT	76.2	(53.5–108.6)	84.9	(52.7–136.7)	65.6	(36.6–117.5)	104.4	(32.1–339.9)
	uRDT	65.3	(47.9–89.1)	68.5	(44.1–106.4)	51.3	(31.5–83.6)	109.7	(40.0–300.7)
LR-	cRDT	0.4	(0.3–0.5)	0.3	(0.2–0.4)	0.3	(0.2–0.4)	0.6	(0.5–0.8)
	uRDT	0.3	(0.3–0.4)	0.3	(0.2–0.5)	0.2	(0.1–0.3)	0.5	(0.3–0.6)
DOR	cRDT	196.1	(125.0–307.8)	279.1	(138.9–561.2)	230.6	(105.6–503.7)	171.2	(51.1–566.9)
	uRDT	203.0	(131.9–312.5)	213.7	(109.7–416.4)	248.8	(115.3–536.9)	242.0	(81.2–714.0)
AUC	cRDT	0.80	(0.77–0.84)	0.85	(0.79–0.90)	0.85	(0.80–0.91)	0.69	(0.63–0.76)
	uRDT	0.84	(0.80–0.87)	0.84	(0.78–0.89)	0.89	(0.84–0.94)	0.77	(0.70–0.84)

*PPV* Positive predictive value, *NPV* negative predictive value, *LR+* positive likelihood ratio, *LR−* negative likelihood ratio, *DOR* diagnostic odds ratio, *AUC* area under the receiver operating characteristic curve

### Parasite densities by RT-qPCR

The GMPD of infections detected by RT-qPCR was 77.5 parasites/µL (95% CI 50.2 to 119.7) and decreased with increasing age. The GMPD was 112.9 parasites/µL (95% CI 65.3 to 195.0) among TP uRDT samples and 145.5 parasites/µL (95% CI 82.0 to 258.0) among TP cRDT samples. FN samples by uRDT or cRDT had GMPDs of < 35 parasites/µL (Table 4).

**Table 4:** Geometric mean parasite densities (GMPDs) by RT-qPCR among uRDT and cRDT positive and uRDT and cRDT negative samples, according to age group

	Result	Method	N	RT-qPCR GMPD (parasites/μL)	95% CI	Min–Max (parasites/μL)
All ages	Positive	RT-qPCR	179	77.5	50.2–119.7	0.6–120,786.2
		uRDT	122	112.9	65.3–195.0	0.6–120,786.2
		cRDT	110	145.5	82.0–258.0	0.6–120,786.2
	Negative	uRDT	57	34.6	17.6–68.2	1.4–40,038.9
		cRDT	69	28.4	15.5–51.9	0.9–40,038.9
< 5-year-olds	Positive	RT-qPCR	63	128.1	57.2–286.8	0.6–120,786.2
		uRDT	43	187.2	69.1–507.2	0.6–120,786.2
		cRDT	44	183.0	69.1–484.6	0.6–120,786.2
	Negative	uRDT	20	56.7	13.7–234.4	1.4–40,038.9
		cRDT	19	56.2	12.5–252.1	1.4–40,038.9
5–< 15 year-olds	Positive	RT-qPCR	64	99.5	47.8–206.9	1.7–59,365.7
		uRDT	51	99.5	42.3–233.9	1.7–59,365.7
		cRDT	46	114.4	45.7–286.6	1.7–59,365.7
	Negative	uRDT	13	99.4	20.9–473.5	5.3–7235.0
		cRDT	18	69.5	19.8–244.5	4.1–7235.0
≥ 15 year-olds	Positive	RT-qPCR	51	32.2	16.2–64.3	0.9–14,991.6
		uRDT	28	65.2	22.5–189.6	0.9–14,991.6
		cRDT	20	152.7	46.0–508.1	2.1–14,991.6
	Negative	uRDT	23	13.7	6.5–29.0	2.4–2311.7
		cRDT	31	11.8	6.1–22.8	0.9–2311.7

**Fig. 4:**
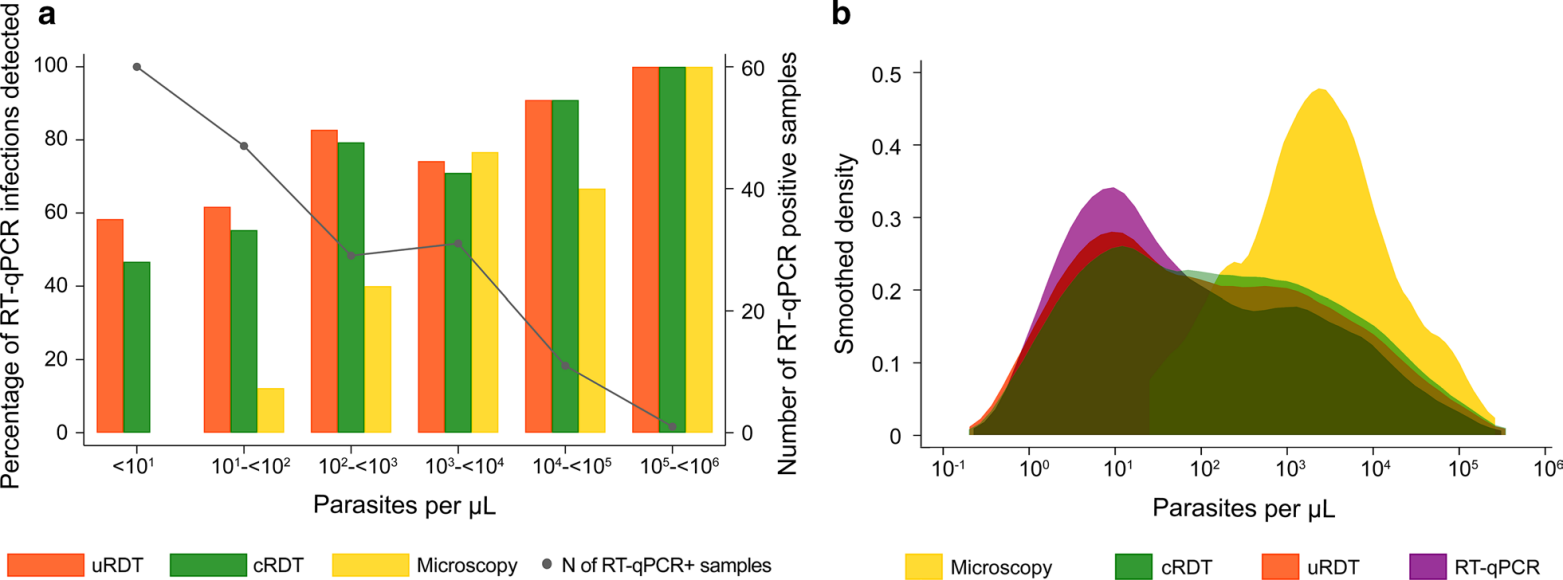
**a** Proportion of RT-qPCR positive infections detected by uRDT, RDT or microscopy per parasite density groups. Gray line: Number of RT-qPCR positives samples per parasite density groups. **b** Parasite density distributions of infections detected by microscopy, cRDT, uRDT and RT-qPCR

The proportion of infections detected by uRDT, cRDT or microscopy increased with increasing parasite density (Fig. 4a). The proportion of infections detected by uRDT and cRDT were similar throughout the parasite density spectrum, while the infections detected by microscopy tended to have higher parasite densities. Figure 4b depicts smoothed parasite density distributions of infections detected by RT-qPCR, uRDT, cRDT and microscopy.

### Antigen detection by ELISA and gene deletions analysis

In order to better understand disagreements between diagnostic tools, 223 samples were analysed by Q-Plex ELISA for different parasite antigens (Table 5). 84.4% (27/32) FP samples by cRDT were HRP2 positive, compared to 80.5% (33/41) of FP by uRDT (p-value = 0.67). The majority of the FP samples that were HRP2-positive were detected among children < 5-years-old (60.6% for uRDTs and 59.3% for cRDTs) and among the 5 to 15 age group (21.7% for uRDTs and 37.0% for cRDTs). HRP2 was also detected in > 70% of samples positive by RT-qPCR but negative by cRDT (73.0%) or by uRDT (70.4%) (Table 5).

**Table 5:** Presence of HRP2, pLDH-Pan, and pLDH-Pf antigen by type of result from RT-qPCR, cRDT and uRDT in 233 samples

			HRP2 positive	pLDH-Pan positive	pLDH-Pf positive
			N = 160/223	N = 114/223	N = 97/223
			n (%)	n (%)	n (%)
RT-qPCR positive N = 134/223	cRDT positive = TP	N = 71	71 (100.0)	56 (78.9)	56 (78.9)
	cRDT negative = FN	N = 63	46 (73.0)	38 (60.3)	33 (52.4)
	uRDT positive = TP	N = 80	79 (98.8)	61 (76.3)	59 (73.8)
	uRDT negative = FN	N = 54	38 (70.4)	33 (61.1)	30 (55.6)
RT-qPCR negative N = 89/223	cRDT positive = FP	N = 32	27 (84.4)	11 (34.4)	8 (25.0)
	cRDT negative = TN	N = 57	16 (28.1)	9 (15.8)	0 (0)
	uRDT positive = FP	N = 41	33 (80.5)	12 (29.3)	8 (19.5)
	uRDT negative = TN	N = 48	10 (20.8)	8 (16.7)	0 (0)

*TP* True positive, *FP* false positive, *TN* true negative, *FN* false negative, *HRP2* histidine-rich protein 2, *pLDH* *Plasmodium* lactate dehydrogenase, *pLDH-Pan* pan specific pLDH, *pLDH-Pf* *P. falciparum* specific pLDH

DNA extraction for the identification of *pfhrp2* and *pfhrp3* gene deletions was successful in 32/55 FN samples by both uRDT and cRDT. Among these, a partial deletion of the exon 2 of the *pfhrp2* gene was found in only one sample (3.1%) (Additional file 2: Figure S1). This sample was negative by both RDTs and by microscopy but had a parasite density of 227.3 parasites/μL measured by RT-qPCR. The antigens HRP2, pLDH-pan and pLDH-*Pf* were also detected in this sample by the antigen assay.

### cRDT and uRDT performance among febrile and afebrile individuals and pregnant women

A secondary analysis was conducted to explore RDTs’ diagnostic performance among febrile and afebrile individuals in the community. An 8.7% (382/4393) of the study population had fever at the time of the study or reported a febrile episode during the preceding 24 h. Using RT-qPCR, malaria was detected in 13.9% (53/382) of these febrile individuals and in 3.1% of afebrile (126/4011) ones. Fever was detected or reported within preceding 24 h in 29.6% (53/179) of the individuals with a positive RT-qPCR, 33.7% (56/166) of individuals with a positive uRDT, and 35.4% (51/144) of individuals with a positive cRDT.

The GMPD of febrile participants was 346.3 parasites/µL (95% CI 152.0 to 788.7) (n = 53) and 41.3 parasites/µL (95% CI 25.6 to 66.5) (n = 126) for afebrile ones. Consequently, the sensitivity of cRDTs was higher among febrile individuals (79.2; 95% CI 65.9 to 89.2) than among afebrile individuals (54.0; 95% CI44.9 to 62.9) (p-value = 0.002). Likewise, the sensitivity of uRDTs was higher among febrile individuals (84.9; 95% CI 72.4 to 93.3) than among afebrile ones (61.1; 95% CI 52.0 to 69.7) (p-value = 0.002). Only the sensitivity among afebrile individuals varied significantly between RDTs.

Two infections were detected by RT-qPCR among the 21 women who reported being pregnant in this study. One infection had a parasite density of 1904 parasites/µL by RT-qPCR and was diagnosed by microscopy, cRDT and uRDT; and had detectable HRP2 antigenaemia. The other infection had a parasite density of 2.9 parasites/µL by RT-qPCR, but was not detected by any other diagnostic tool despite having detectable HRP2. Neither presented with a fever during the survey nor reported having had a fever or taking anti-malarials in the preceding 30 days.

## Discussion

This study evaluated the performance of a recently developed ultrasensitive malaria RDT in field conditions among individuals in a malaria endemic community of southern Mozambique. The study showed that the sensitivity of the Abbott *Pf* malaria uRDT (68.2) was marginally higher compared to the SD-Bioline *Pf* malaria cRDT (61.5), while specificities were very similar (99.0 for uRDT *vs.* 99.2 for cRDT). This translated into very few differences in absolute numbers. The performance of both types of RDTs was affected by age in a study population exposed to malaria since a very young age [47]. In this context, both uRDTs and RDTs performed best in 5–< 15 year-olds, and worse in > 15 year olds. There was barely a difference between the parasite densities of infections detected by either test and both RDTs detected a similar proportion of HRP2-positive samples. 31.8% (57/179) and 38.5% (69/179) of infections were missed by uRDT and cRDT, respectively, which was not driven by *pfhrp2* or *pfhrp3* deletions, as only one FP sample was detected with a partial *pfhrp2* deletion, in agreement with previous findings from Mozambique [31, 32].

Overall, these results raise the question of the added value of the uRDTs evaluated in this study, in Mozambique. In fact, the utility of these uRDTs remains unclear, partly as a result of the emergent body of evidence in line with the performance outcomes presented here [21, 26, 27, 29]. In summary, three main use-cases have been proposed for this diagnostic tool: (1) To be used in malaria elimination contexts to more accurately measure prevalence or guide test-and-treat strategies to interrupt malaria transmission in the community, (2) for improved case management, and 3) to detect low-density infections among pregnant women. This study provides evidence to inform the first use-case, and partially discuss the second and third.

uRDTs have been proposed as a tool to detect malaria infections even at low-density infections (below 100–200 parasites/µL), thus triggering their treatment in order to interrupt transmission in areas aiming for elimination, through mass test and treat (MTAT) campaigns, or proactive or reactive testing and treating. MTAT strategies evaluated throughout Africa and Asia have to date shown not to have a strong impact on malaria transmission when using cRDTs [23, 48–52], and consequently, the WHO does not recommend it as a tool to reduce transmission [53]. However, it was argued that the impact of MTAT could be different if conducted using a highly sensitive RDT. Nevertheless, the field-based sensitivity of the uRDTs reported here and in other studies is similar to that of cRDTs, and mathematical modeling suggests that with the current uRDT performance, an impact on transmission with MTAT would only be observed if > 85% of the target population is treated [24]. This is a very high effective coverage that is rarely achieved in the community [54], and is arguably cost-effective. Additionally, other more cost-effective interventions such as mass drug administration (MDA) have already shown to have a strong impact on transmission in endemic areas [35, 55–57] irrespective of subpatent infection detection in the community. However, MDAs do not lead to the full interruption of malaria transmission [35] and strategies to target the remaining foci of transmission are still unclear. Highly sensitive RDTs have also been proposed to have a role in reactive strategies. Nevertheless, evidence against this option has already emerged from Cambodia where uRDTs failed to show significant improvement in diagnostic performance over cRDTs when used for active case detection, and other studies evaluating this question in other areas may be needed [26].

This study showed that the sensitivity of both RDTs significantly dropped among individuals older than 15 years-old, particularly for cRDTs (39.2) compared to uRDTs (54.9) due to the high prevalence of lower density infections in this age group likely as a result of the cumulative malaria exposure [58]. Moreover, circulating host antibodies against HRP2 could have interfered with HRP2 detection by both RDTs and contributed to performance differences by age group [59, 60]. This points out a potential utility of uRDTs to detect infections among the semi-immune adult population in the community, although in absolute terms this translated into 8 additional infections detected by the uRDTs among participating adults. Nevertheless, this finding has not been consistently observed, as a similar study conducted in The Gambia reported a lower sensitivity of the same uRDT among individuals older than 10 (< 43.7%) compared to the sensitivity presented here. They also reported that the uRDT sensitivity was lowest in children < 5 years old, although the study did not measure parasite densities or HRP2 levels [24]. In any case, the question remains as to whether the detection of subpatent infections is required to ultimately interrupt transmission in a malaria endemic area.

uRDTs have also been proposed to improve malaria case management at the health facility level [27]. This study revealed that the sensitivity of both RDTs increased among febrile individuals, to 84.9 for uRDTs and 79.2 for cRDTs. This indicates that febrile cases which usually suffer from higher density infections as shown in this study (GMPD of 346.3 parasites/µL), are likely to be rightly diagnosed with both types of RDTs in southern Mozambique [32]. Therefore, this suggests that uRDTs do not provide added benefits to the detection of infections among febrile individuals in the community when compared to regular RDTs, a finding that has been similarly observed among febrile outpatients in Tanzania [27]. Similarly, studies conducted among pregnant women observed a higher performance of uRDTs among afebrile women compared to febrile ones [28, 30]. These studies were conducted under laboratory conditions using retrospectively collected samples [28–30]. However, there is currently no evidence of the performance of uRDTs among pregnant women when the tests are directly conducted at point of care. Acknowledging that this study was not powered nor designed to answer this question, it is worth noting that among the two afebrile infections detected by RT-qPCR in pregnant women, cRDTs and uRDTs only detected the high-density infection, but not the low-density one, despite the presence of HRP2 in both samples.

One of the commonly reported concerns of using a more sensitive HRP2-based RDT is that it may provide a higher number of FP in post-treated individuals, due to its capacity to detect remaining concentrations of HRP2 in the blood for a longer period of time [27]. This is the first report of the application of an antigenic ELISA applied to dried blood spots collected directly from the field. Using this technique, more than 80% of FP samples were HRP2-positive, but uRDTs did not detect a significantly larger number of FP results as a product of the increasing level of HRP2 detection of the test. The fact that there were ~ 20% of HRP2-negative samples that were falsely positive by cRDT or uRDT could be explained by the limit of HRP2 detection of the ELISA technique used. While the limit of detection on whole blood pellets is significantly lower than that of the uRDT [61, 62], the performance of the ELISA on DBS is likely to be lower depending on the efficiency of antigen recovery and the antigen stability on the DBS (Jang et al. 2020; pers. commun,). Consequently, the uRDT may have detected true HRP2 positive cases with low HRP2 concentration that were missed by the ELISA. With the advent of novel highly sensitive malaria antigen assays, understanding their performance in low parasite density specimens will be critical to accurately interpret its results in sub-microscopic infections [63, 64].

This study is subject to several limitations. First, the diagnostic performance analysis was conducted using RT-qPCR as the gold standard. In the younger age groups, the proportion of positive RT-qPCR samples was very similar or even lower to the proportion of positive uRDT samples. This can be partly explained by the high number of FP samples that were HRP2-positive in younger age groups. Children may be more likely to remain RDT positive for a longer time after anti-malarial treatment than adults, as a result of less acquired immunity and higher parasite densities leading to higher HRP2 circulating concentrations [6, 59]. However, it is also possible that the level of detection of the RT-qPCRs used in this study may have missed some infections. This may have been the case given that the sensitivity and specificity of uRDT reported here are slightly higher than those reported in other malaria endemic areas [23–26]. Similarly, the presence of pLDH antigens among RT-qPCR negative samples indicate that some of these samples may have been positive, although these results may also be explained by the limitations of the ELISA assay for this antigen as mentioned above. Additionally, RDT results could have also been affected by factors such as the prozone effect, which could have been the cause of high-density infections not detected by either RDT [11]. In spite of intense training, field workers not following standard procedures (e.g. not collecting the right amount of blood, not placing the right amount of reagent, not waiting for a sufficiently long period of time) or even data entry errors may have compromised the quality of these results [65].

## Conclusion

Overall, these results contributed to the growing evidence from field-based studies that show that the Abbott *Pf* malaria uRDTs do not substantially outperform SD-Bioline *Pf* malaria RDTs in the community or among febrile cases, and are still not comparable to molecular methods like RT-qPCRs. Therefore, the use-cases for the currently available uRDTs should be reviewed before malaria endemic countries with a long experience using the standard RDTs decide to switch to a different yet similarly performing test.

## Supplementary Information

**Additional file 1: Table S1.** Definitions of performance indicators used. TP = True positive, FP = False positive, TN = True negative, FN = False negative. **Table S2.** Study population characteristics, overall and by region

**Additional file 2: Figure S1.** Flow-chart of the parasite pfhrp2 and pfhrp3 gene deletions analysis

## Data Availability

The datasets generated during and/or analysed during the current study are not publicly available due to the agreements reached with the regulatory authorities of the country but are available from the corresponding author on reasonable request.

## Abbreviations

AL: Artemether–lumefantrine
AUC: Area under the receiver operating characteristic curve
CI: Confidence interval
CISM: Centro de Investigação em Saúde de Manhiça (Manhiça Health Research Center)
cRDT: Conventional rapid diagnostic test
DBS: Dried blood spot
DOR: Diagnostic odds ratio
DSS: Demographic surveillance system
FN: False negative
FP: False positive
ELISA: Enzyme-linked immunosorbent assay
GMPD: Geometric mean parasite density
HRP2: Histidine-rich protein 2
IQR: Interquartile range
IRS: Indoor residual spraying
ITN: Insecticide-treated net
LLINs: Long lasting insecticidal nets
LR+: Positive likelihood ratio
LR−: Negative likelihood ratio
MDA: Mass Drug Administration
MTAT: Mass test and treat
NPV: Negative predictive value
OR: Odds ratio
PCR: Polymerase chain reaction
P.f: *Plasmodium falciparum*
pLDH: *Plasmodium* Lactate dehydrogenase
pLDH-Pan: Pan specific pLDH
pLDH-Pf: *Plasmodium falciparum* Specific pLDH
PPV: Positive predictive value
RDT: Rapid diagnostic test
ROC: Receiver-operating characteristic
RT-qPCR: Real-time quantitative polymerase chain reaction
TN: True negative
TP: True positive
uRDT: Ultrasensitive rapid diagnostic test
WHO: World Health Organization
