# Supplementary material for: Field performance of ultrasensitive and conventional malaria rapid diagnostic tests in southern Mozambique
Source: Malar J. 2020 Dec 7;19:451. doi: 10.1186/s12936-020-03526-9 (PMC7720469; doi:10.1186/s12936-020-03526-9)
Supplement: Supplementary file 1 — Additional file 1: Table S1. Definitions of performance indicators used. TP = True positive, FP = False positive, TN = True negative, FN = False negative. Table S2. Study population characteristics, overall and by region [file 12936_2020_3526_MOESM1_ESM.docx]

**TITLE:** Field performance of ultrasensitive and conventional malaria rapid diagnostic tests in southern Mozambique

**ADDITIONAL TABLES**

**AUTHORS**

Beatriz Galatas*^1,2^, Alfredo Mayor^1,2,3^, Himanshu Gupta^1a^, Núria Balanza^1^, Ihn Kyung Jang^4^, Lidia Nhamussua^2^, Wilson Simone^2^, Pau Cisteró^1^, Arlindo Chidimatembue^2^, Humberto Munguambe^2^, Francisco Saute^2^, Pedro Aide^2,5^, Quique Bassat^1,2,3,6,7^

*Corresponding author: [beatriz.galatas@isglobal.org](mailto:beatriz.galatas@isglobal.org)

**AFFILIATIONS**

1 ISGlobal, Hospital Clínic - Universitat de Barcelona, Barcelona, Spain

2 Centro de Investigação em Saúde de Manhiça, Maputo, Mozambique

3 CIBER Epidemiología y Salud Pública (CIBERESP), Madrid, Spain

4 PATH, Seattle, USA

5 National Institute of Health, Ministry of Health, Maputo, Mozambique

6 ICREA, Pg. Lluís Companys 23, 08010 Barcelona, Spain

7 Pediatric Infectious Diseases Unit, Pediatrics Department, Hospital Sant Joan de Déu (University of Barcelona), Barcelona, Spain

^a^ Current address: Department of Infection Biology, Faculty of Infectious and Tropical Diseases, London School of Hygiene and Tropical Medicine, London, UK

**Table S1. Definitions of performance indicators used.** TP = True positive, FP = False positive, TN = True negative, FN = False negative.

| INDICATOR | DEFINITION |
| --- | --- |
| Sensitivity | Probability of getting a positive test result in infected subjects:  *TP / TP + FN* |
| Specificity | Probability of a negative test result in uninfected subjects:  *TN / TN + FP* |
| Positive predictive value (PPV) | Probability of being infected with a positive test result:  *TP / TP + FP* |
| Negative predictive value (NPV) | Probability of not being infected with a negative test result:  *TN / TN + FN* |
| Positive likelihood ratio (LR+) | Likelihood of a positive RDT occurring in infected subjects compared to uninfected subjects:  *Sensitivity / 1 – Specificity* |
| Negative likelihood ratio (LR-) | Likelihood of a negative RDT occurring in infected subjects compared to uninfected subjects:  *1 – Sensitivity / Specificity* |
| Diagnostic odds ratio (DOR) | Ratio of the odds of positivity in subjects with disease relative to the odds in subjects without disease:  *LR+ / LR-* |
| Area under the receiver operating characteristic curve (AUC) | *Sensitivity + Specificity / 2* |
| Probability of having a positive RDT among the infections detected by PCR | *Number of positive RDTs (uRDT or cRDT) / Number of positive RT-qPCR tests x 100* |

**Table S2: Study population characteristics, overall and by region**

|  | All regions  (N=4,396) | Magude  (N=3,349) | Manhiça  (N=684) | Xinavane  (N=363) |
| --- | --- | --- | --- | --- |
|  | n (%*) | n (%*) | n (%*) | n (%*) |
| Sex (N [% missing]) | **4,353 (1.0)** | **3,306 (1.3)** | **684 (0)** | **363 (0)** |
| Male | 1,951 (44.8) | 1,516 (45.9) | 281 (41.1) | 154 (42.4) |
| Female | 2,402 (55.2) | 1,790 (54.1) | 403 (58.9) | 209 (57.6) |
| Age (N [% missing]) | **4,381 (0.3)** | **3,335 (0.4)** | **683 (0.2)** | **363 (0)** |
| <5 | 2,372 (54.1) | 1,849 (55.4) | 313 (45.8) | 210 (57.9) |
| 5-<15 | 1,159 (26.5) | 963 (28.9) | 120 (17.6) | 76 (20.9) |
| ≥15 | 850 (19.4) | 523 (15.7) | 250 (36.6) | 77 (21.2) |
| Pregnancy in women between 12-49 years old^§^ (N [% missing]) | **302 (40.3)** | **223 (33.1)** | **54 (53.9)** | **25 (54.6)** |
| Yes | 21 (7.0) | 16 (7.2) | 5 (9.3) | 0 (0) |
| No | 255 (84.7) | 187 (84.2) | 47 (87.0) | 21 (84.0) |
| Doesn’t know | 25 (8.3) | 19 (8.6) | 2 (3.7) | 4 (16.0) |
| Fever at visit (N [% missing]) | **4,128 (6.1)** | **3,105 (7.3)** | **666 (2.6)** | **357 (1.7)** |
| Yes | 19 (0.5) | 10 (0.3) | 1 (0.2) | 8 (2.2) |
| No | 4,109 (99.5) | 3,095 (99.7) | 665 (99.9) | 349 (97.8) |
| Febrile episode in preceding 24h (N [% missing]) | **4,387 (0.2)** | **3,345 (0.1)** | **681 (0.4)** | **361 (0.6)** |
| Yes | 371 (8.5) | 298 (8.9) | 49 (7.2) | 24 (6.7) |
| No | 3,963 (90.3) | 3,004 (89.8) | 630 (92.5) | 329 (91.1) |
| Doesn’t know | 53 (1.2) | 43 (1.3) | 2 (0.3) | 8 (2.2) |
| Febrile episode in preceding month (N [% missing]) | **4,394 (0.1)** | **3,348 (0.03)** | **683 (0.2)** | **363 (0)** |
| Yes | 640 (14.6) | 494 (14.8) | 91 (13.3) | 55 (15.2) |
| No | 3,693 (84.1) | 2,799 (83.6) | 588 (86.1) | 306 (84.3) |
| Doesn’t know | 61 (1.4) | 55 (1.6) | 4 (0.6) | 2 (0.6) |
| Antimalarials uptake in preceding month (N [% missing]) | **4,342 (1.2)** | **3,305 (1.3)** | **680 (0.6)** | **357 (1.7)** |
| Yes | 284 (6.5) | 252 (7.6) | 30 (4.4) | 2 (0.6) |
| No | 4,058 (93.5) | 3,053 (92.4) | 650 (95.6) | 355 (99.4) |
| Doesn’t know | 0 (0) | 0 (0) | 0 (0) | 0 (0) |
| ITN use in preceding night (N [% missing]) | **4,371 (0.6)** | **3,324 (0.8)** | **684 (0)** | **363 (0)** |
| Yes | 3,218 (73.6) | 2,497 (75.1) | 438 (64.0) | 283 (78.0) |
| No | 1,149 (26.3) | 827 (24.9) | 242 (35.4) | 80 (22.0) |
| Doesn’t know | 4 (0.1) | 0 (0) | 4 (0.6) | 0 (0) |
| Household IRS in preceding year (N [% missing]) | **3,990 (9.2)** | **2,943 (12.1)** | **684 (0)** | **363 (0)** |
| Yes | 2,935 (73.6) | 2,274 (77.3) | 384 (56.1) | 277 (76.3) |
| No | 1,034 (25.9) | 669 (22.7) | 287 (42.0) | 78 (21.5) |
| Doesn’t know | 21 (0.5) | 0 (0) | 13 (1.9) | 8 (2.2) |

§ Total women between 12-49 years old: All regions N=504, Magude N=332, Manhiça N=117***,*** Xinavane N=55

*Proportion calculated out of the non-missing values.

ITN = Insecticide-treated net
