# Supplementary figures and images for: Field performance of ultrasensitive and conventional malaria rapid diagnostic tests in southern Mozambique
Source: Malar J. 2020 Dec 7;19:451. doi: 10.1186/s12936-020-03526-9 (PMC7720469; doi:10.1186/s12936-020-03526-9)

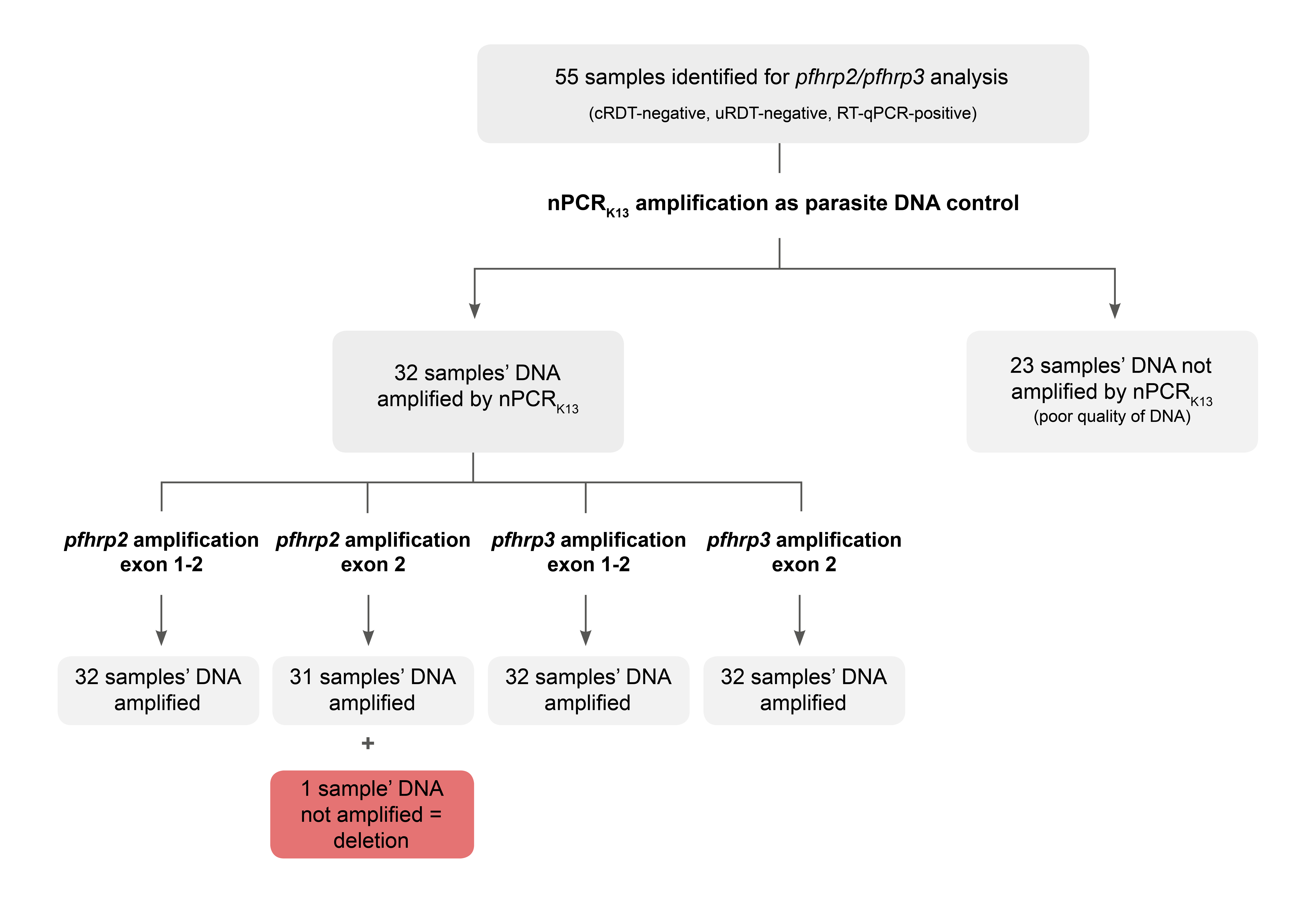

Supplement: Supplementary file 2 — Additional file 2: Figure S1. Flow-chart of the parasite pfhrp2 and pfhrp3 gene deletions analysis [file 12936_2020_3526_MOESM2_ESM.jpg]
